# Measurements of fetal dose with Mevion S250i proton therapy system with HYPERSCAN

**DOI:** 10.1002/acm2.13957

**Published:** 2023-04-12

**Authors:** Karolyn M. Hopfensperger, Xing Li, Adam Paxton, Vikren Sarkar, Fan‐Chi Frances Su, Ryan G. Price, Bill Salter, Sara St. James

**Affiliations:** ^1^ Department of Radiation Oncology University of Kansas Medical Center Kansas City Kansas USA; ^2^ Department of Radiation Oncology Huntsman Cancer Institute University of Utah Salt Lake City Utah USA

**Keywords:** fetal dose, scanning beam proton therapy

## Abstract

**Purpose:**

To characterize potential dose to the fetus for all modes of delivery (dynamic adaptive aperture, static adaptive aperture, and no adaptive aperture) for the Mevion S250i Proton Therapy System with HYPERSCAN and compare the findings with those of other available proton systems.

**Materials and Methods:**

Fetal dose measurements were performed for all three modes of dose delivery on the Mevion S250i Proton therapy system with HYPERSCAN (static aperture, dynamic aperture and uncollimated). Standard treatment plans were created in RayStation for a left‐sided brain lesion treated with a vertex field, a left lateral field, and a posterior field. Measurements were performed using WENDI and the RANDO with the detector placed at representative locations to mimic the growth and movement of the fetus at different gestational stages.

**Results:**

The fetal dose measurements varied with fetus position and the largest measured dose was 64.7 μSv per 2 Gy (RBE) fraction using the dynamic aperture. The smallest estimated fetal dose was 45.0 μSv per 2 Gy (RBE) at the base of the RANDO abdomen (47 cm from isocenter to the outer width of WENDI and 58.5 cm from the center of the WENDI detector) for the static aperture delivery. The vertex fields at all depths had larger contributions to the total dose than the other two and the dynamic aperture plans resulted in the highest dose measured for all depths.

**Conclusion:**

The reported doses are lower than reported doses using a double‐scattering system. This work suggests that avoiding vertex fields and using the static aperture will help minimize dose to the fetus.

## 1 INTRODUCTION

Proton therapy is an established form of radiation therapy treatment available to patients throughout the world and ∼40 centers currently offer proton therapy treatment to patients in the United States.^1^ Many patients may benefit from the finite range and reduced exit dose, distinct characteristics of the radiation dose deposition in proton therapy. The reduced exit dose allows for treatment plans to be created for patients such that nearby, at‐risk healthy tissues and organs may be spared, receiving very little radiation dose from protons.

Out‐of‐field dose is a concern when treating young patients who may develop secondary cancers, cardiac dose, and in the treatment of pregnant patients. In proton radiation therapy the out‐of‐field dose to patients is predominantly from neutrons and may originate from the equipment or from interactions within the patient.^2^ For the estimate of out‐of‐field dose, measurements of the neutron dose remain the gold standard.^3^ This work focuses on aspects related to estimating the dose to the fetus.

AAPM Task Group Report #36 includes a summary of risk as a function of the dose to the fetus and reports that below 0.05 Gy, there is little risk of damage. The report of AAPM Task Group 36 has also been used to estimate fetal dose from photon therapy but there is not a comprehensive reference or guideline for patients receiving proton radiation therapy. Some of the information in this report can be applied to proton therapy but there still exists a gap between fetal dose estimates for photon therapy and proton therapy, and guidelines for best practices.^4^ Similar to photons, the choice of beam angles and treatment technique can mitigate the total dose delivered to a fetus. Unlike in photons, the estimated fetal dose from protons can have a large dependency on the treatment delivery system.

As the technology for proton therapy has advanced, the treatment delivery techniques have changed from using scattering systems and external brass apertures to shape the proton field to magnetically steering the proton beam, as is done in pencil beam scanning (PBS) systems.^5^ Apertures may be used in conjunction with PBS, as proposed by Dowdell et al.^6^ and further characterized by Maes et al.^6^ In these scenarios, custom brass apertures designed to collimate the edges of the proton radiation field decrease the lateral penumbra and have the potential to provide improved sparing of organs at risk. Multi‐leaf collimators have also been used to shape pencil beam scanning proton fields and are extensively discussed in Hyer et al.^7^ On the Mevion S250i, the lateral edges of the field may be collimated with an adaptive aperture. This aperture is inside the nozzle and is comprised of seven nickel collimators. Unlike patient‐specific brass apertures, the adaptive aperture has the capability to change shapes with energy layers, allowing for the collimation to change with depth.^8^

This work investigates the out‐of‐field dose from neutrons on the Mevion S250i single‐room proton therapy system with HYPERSCAN^6^ in the context of fetal dose estimates, and to our knowledge represents the first published estimates of fetal dose with the system, including an evaluation of all collimation techniques available. This is of particular interest as the system described combines pencil beam scanning with several methods of collimating the beam using adaptive apertures. The results are compared to published results on other systems, adding to the base of clinical knowledge of out‐of‐field dose in proton radiation therapy.

## 2 METHODS

In this work, an anthropomorphic phantom with a large intracranial mass was used as the model patient. Treatment plans were created using a CT scan of the phantom and measurements of the out‐of‐field dose were performed on the clinical system. Three equivalent treatment plans were created using the available collimation techniques on the Mevion S250i. These collimation techniques include:

1. Without an adaptive aperture (e.g., the proton beam locations are magnetically steered without the use of apertures).
2. With a static aperture (e.g., the HYPERSCAN aperture which is internal to the Mevion S250i was shaped to the largest projection of the target plus a margin of 0.7 cm).
3. With a dynamic aperture (e.g., the Hyperscan aperture changes shape and position for each delivered layer of the dose distribution, with a lateral margin for spot spacing of 0.4 cm).

### 2.1 Treatment planning

Treatment plans were generated using Monte Carlo optimization in RayStation version 11A (RaySearch Laboratories, Stockholm, Sweden) to deliver 66 Gy(RBE) in 2 Gy(RBE) fractions to a left‐sided spherical target of 6 cm diameter. Each treatment plan had the same three treatment fields, including a left lateral field, a posterior field, and a vertex field. The Monte Carlo dose calculation used does not model secondary neutrons and cannot be used to estimate out‐of‐field dose.

**FIGURE 1:**
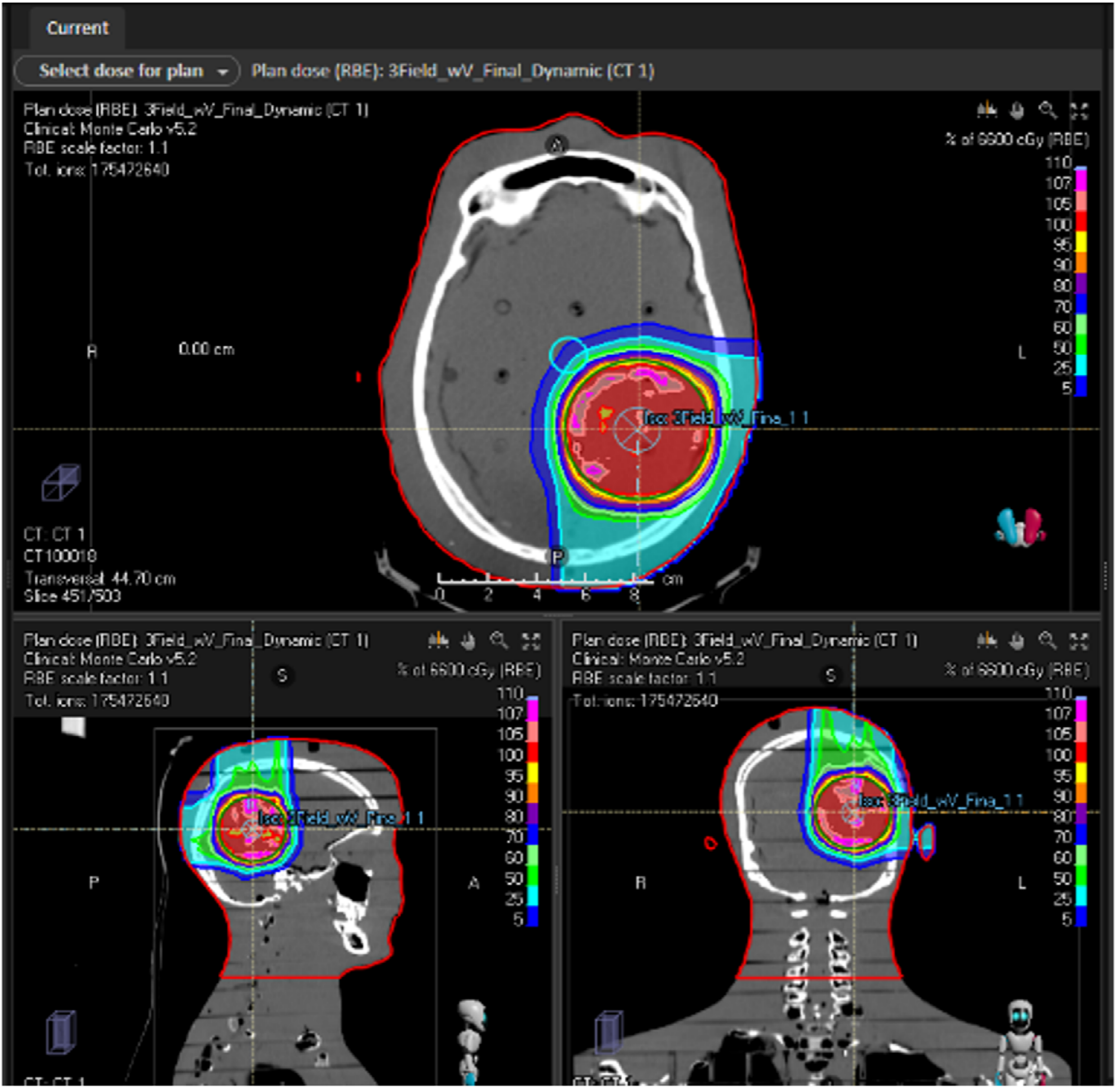
Dose distribution for 6 cm diameter left lateral lesion using vertex, posterior, and left lateral fields.

**FIGURE 2:**
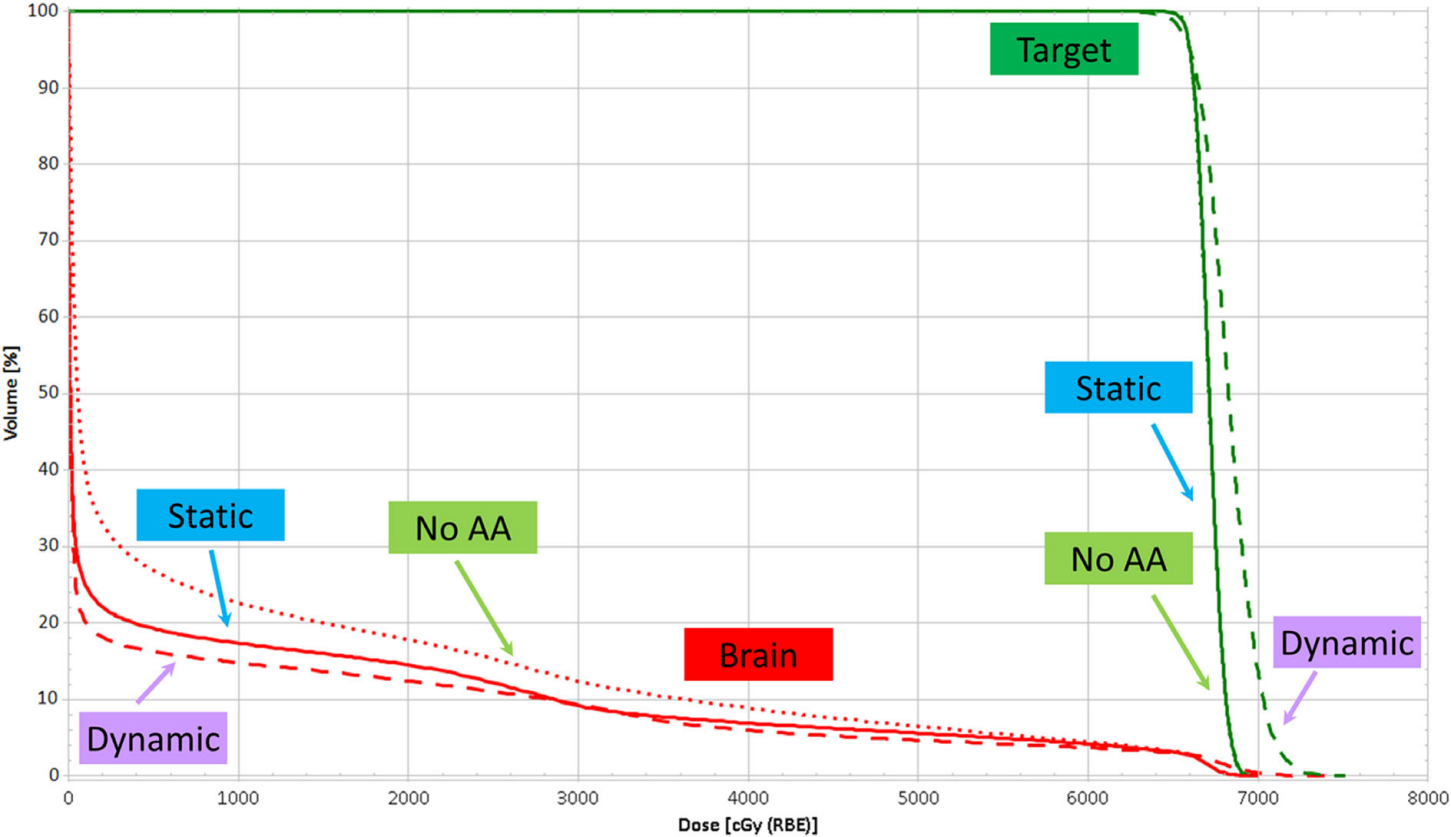
Dose volume histograms for static aperture, dynamic aperture, and uncollimated treatments. Normal brain and target are shown.

Treatment plans were created using CT scans of the RANDO phantom. The plan dose distribution is shown in Figure 1 for the scenario where the dynamic apertures were used, and the dose‐volume histograms for all three treatments are shown in Figure 2. Three plans were created corresponding to the three different collimation techniques: dynamic adaptive aperture, static adaptive aperture, and uncollimated (“no AA”). All plans had 95% of the target volume treated to the prescription dose. Dose deposition differences inherent to the three collimation techniques are evident on the dose‐volume histogram. The dose to normal tissue (brain structure) is less in the scenarios where apertures are used (both static and dynamic) and is lowest when the dynamic aperture is used. The dose to the target is less uniform for the scenario where the dynamic aperture is used, resulting in larger high‐dose regions within the target. While Monte Carlo optimization and dose calculation is used for all treatment plans, the treatment planning software does not provide information about the out‐of‐field dose at the investigated positions.

### 2.2 Neutron measurements

**FIGURE 3:**
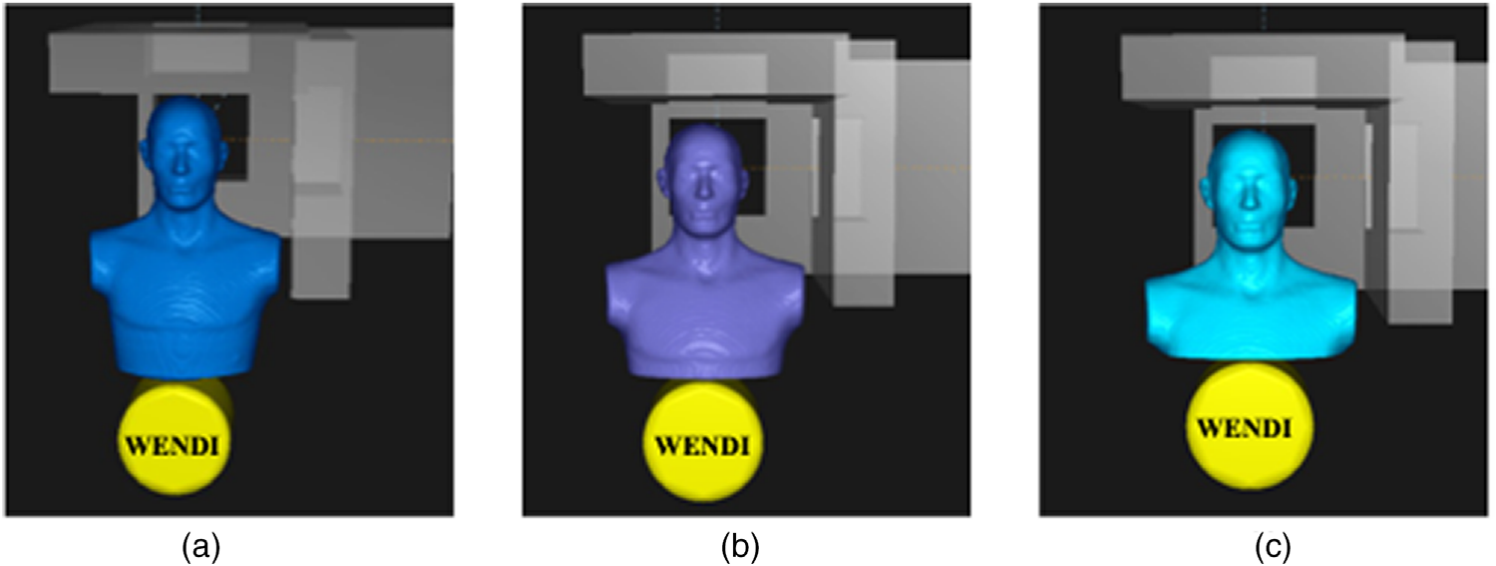
Illustration of the RANDO phantom and the positions of the WENDI for neutron dose equivalent measurement.

All measurements were performed using WENDI (Thermo Fisher Scientific, Waltham, MA) and the RANDO anthropomorphic phantom (Radiology Support Devices, Inc., Long Beach, CA) with the detector placed at representative locations to mimic the growth and movement of the fetus at different gestational stages. The WENDI detector was placed at three different superior to inferior locations, with the locations separated by 7.5 cm of phantom material, as shown in Figure 3. At each measurement location, the detector was moved and 7.5 cm of phantom was removed, ensuring that the measurements were representative of the differences in tissue that arise over the course of a pregnancy. The measurement positions were 58.5 cm from isocenter (66.5 cm from the most superior point of the anthropomorphic phantom) to the center of the WENDI detector (which is 47 cm from the superior width of WENDI), 51 cm from isocenter (39.5 cm from WENDI edge), and 43.5 cm from isocenter (32 cm from WENDI edge). Measurements for each field were repeated three times, for a total of nine measurements per plan. The WENDI measurement is the neutron ambient dose equivalent H*(10), accounting for the physical dose deposited and the quality factor (Q).^9^ Bubble detector measurements using BD‐PND detectors (Bubble Technology Industries, Chalk River, ON) with a sensitivity of 0.12–0.15 bubbles/μSv at 52 cm from isocenter were used to confirm the measurements taken with the WENDI detector for the adaptive aperture plan, which is the plan for which the neutron dose was expected to be highest.

### 2.3 Linac measurements

To characterize how the Mevion S250i proton out‐of‐field dose compares to a modern (6 MV) VMAT treatment plan for a similar target volume, a dosimetrically similar plan was created using the Eclipse Treatment Planning System (Varian Medical Systems, Palo Alto, CA) employing a Varian Edge accelerator. Out‐of‐field contribution was measured using optically stimulated luminescent detectors (OSLDs). As in the proton plan, the prescription dose was 66 Gy in 33 fractions (2 Gy per fraction) to the 6 cm target volume and the plan was optimized to provide desired coverage of the PTV (95% of the PTV received the full prescription dose). The photon plans were delivered on a Varian Edge linear accelerator. OSLDs were placed at the same positions as the edge of the WENDI (47, 39.5, and 32 cm from isocenter). The OSLDs were annealed prior to treatment to ensure that the residual dose was minimized and the fields were repeated five times to ensure an adequate measurement signal.

## 3 RESULTS

**FIGURE 4:**
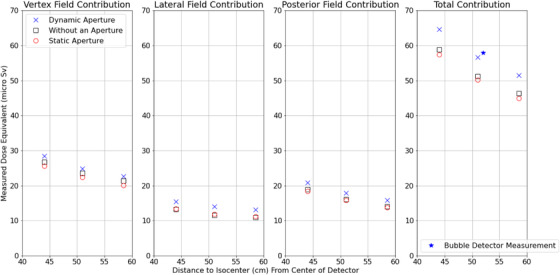
Measurements of the out of field contribution. The blue cross is for the dynamic aperture measurements, the black square represents the measurements where an aperture was not used, and the red circle represents measurements where the apertures were static. The bubble detector measurement for the adaptive aperture field at 52 cm from isocenter is shown as a star. The error bars to demonstrate standard deviation are too small in comparison to the size of the dose measurements.

Fetal dose measurements varied with detector position, and the largest measured dose was 64.7 μSv (±0.2 μSv) per 2 Gy(RBE) using the dynamic aperture. The smallest estimated fetal dose was 45.0 μSv (±0.2 μSv) per 2 Gy(RBE) at the base of the RANDO abdomen (35.5 cm from isocenter) for the static aperture delivery. The vertex fields at all depths had larger contributions to the total fetal dose than the other two fields. The dynamic aperture plans resulted in the highest fetal dose measured for all depths. The contributions measured from the individual beams and the three‐beam plans for all measurement points are shown in Figure 4. The out‐of‐field dose for the photon plan per 2 Gy treatment delivered is shown in Table 1.

**TABLE 1:** VMAT measurements with an anthropomorphic phantom and OSLDs.

OSLD location from ISO (cm)	OSLD depth (cm)	Average reading (cSv) and range of values (cSv)
32	1.5	0.661 (0.644–0.677)
32	6	0.530 (0.519–0.542)
39.5	1.5	0.290 (0.278–0.305)
39.5	6	0.302 (0.300–0.304)
47	1.5	0.171 (0.163–0.178)
47	6	0.163 (0.159–0.167)

## 4 DISCUSSION

Estimated fetal doses ranged from 45.0 μSv (±0.2 μSv) to 64.7 μSv (±0.2 μSv) per 2 Gy(RBE). As expected, the vertex fields for each beam collimation mode contributed the highest dose to the fetus. Like photon fields, this suggests that when treating a pregnant patient's brain, vertex fields should be avoided if possible, to limit the dose to the fetus. Our data also demonstrates that the use of the Mevion system's Adaptive Aperture in “Static Aperture Mode” is likely to minimize out‐of‐field dose for similar beam arrangements. Increased dose from neutrons produced in the patient is a source of the higher dose from the plan with no adaptive aperture present. All of the measurements in this work demonstrate that the total out‐of‐field dose to a fetus would be significantly less than the 0.05 Gy value listed in TG‐36.

There is concern regarding dose pile‐up in the WENDI detector, causing underestimation of neutron dose, and previous studies have shown that WENDI will underestimate dose from a Mevion system.^10^, ^11^ As a result of these studies, bubble detectors, which have no such issue with dose pile‐up, were used to confirm the results of the measurements. The average dose for the bubble detectors measured location to isocenter was 57.9 μSv per 2 Gy(RBE), consistent with the other measurements in this study. Bubble detectors on their own have additional limitations, as discussed in depth in AAPM TG‐158.^12^ The purpose of including these measurements here was to confirm that the other measurements in the study were not limited by detector dead time.

For a dosimetrically similar VMAT plan, the out‐of‐field dose was significantly higher with photons than with protons. This is due to scattered radiation and not due to neutrons, as a 6 MV VMAT plan would have almost no neutron dose, while a proton plan would have a much higher neutron dose. The out‐of‐field dose could be minimized in this scenario by treating this volume with a 3D photon plan instead of a VMAT plan, but this would result in a less conformal treatment plan. The out‐of‐field dose for the VMAT plan is presented here as an initial reference point for out‐of‐field dose in a VMAT treatment plan scenario. The over‐response of detectors due to energy dependence is discussed in the report of AAPM TG 158,^12^ with OSLDs having an over‐response in the range of 5%–13%. In the VMAT treatment plan, the cumulative dose to the fetus would range from approximately 5 cGy for 33 fractions (using the largest distance to the isocenter and the measurement depth of 6 cm) to 17.49 cGy for 33 fractions (using the smallest distance to isocenter and the depth of 6 cm). All these measurements would exceed the TG‐36 recommendation of 5 cGy; the treatment plan could be intentionally varied to decrease the out‐of‐field dose.

In comparison to other proton treatment modalities, the neutron dose equivalent for the Mevion pencil beam scanning system is equivalent or reduced. The 32.35 μSv per Gy(RBE), measured here represents a significant decrease from the 2.59–3.95 mSv/Gy equivalent measured by Howell et al. for Mevion double scattering system.^8^ Heimovaara et al. showed that for a nasopharyngeal carcinoma treated with protons, the estimated total dose to a fetus was 5.5 mSv, with 4.6 mSv from neutrons alone, at a distance of 20 cm from the border of the CTV and at 7 cm depth.^13^ Additional work by Knežević et al. demonstrated the differences in dose for proton therapy compared to 3D conformal radiation therapy, IMRT, and Gamma Knife (Elekta, Stockholm, Sweden) with the proton therapy plan containing two coplanar beams treating a brain lesion in a pediatric phantom.^14^ The study found a decreased total organ dose equivalent for intensity modulated proton therapy (IMPT) with pencil beam scanning compared to photon therapy.^14^ Compared to phantom measurements taken with bubble detectors at the Indiana University Cyclotron double scattering system, reported by Mesoloras et al., the fetal dose is reduced from the 25–871 μSv per Gy to the maximum 64.7 μSv per 2 Gy(RBE) reported here.^15^ With previous literature demonstrating that passively scattered proton therapy is not associated with a significant increase in secondary malignancies when compared with photon therapy,^3^, ^16^ our work suggests that this risk is further reduced when using pencil beam scanning systems, such as the Mevion S250i system. In all scenarios, however, the cumulative dose over 33 fractions would be far less than the TG‐36 quoted value (0.02 mSv for the entire course compared to 0.05 Gy). Hälg and Schneider compiled a list of out‐of‐field dose measurements for other pencil beam scanning systems, and neutron dose equivalents can vary widely depending on energy and measurement distance from the isocenter. However, the measurements in our study are within the range of the pencil beam scanning measurements reported (1.2–79 μSv per Gy).^17^

In comparison to other studies on proton fetal dose, this work demonstrates that the Mevion pencil beam scanning system has slightly higher out‐of‐field dose measurements than other pencil beam scanning systems, but that the estimated dose to the fetus is still lower than that for double scattering proton therapy.^15^, ^18^, ^19^ Side by side comparisons between systems remain challenging as differences in the delivered treatment plan, measurement conditions and other geometric factors (e.g., beam angles, measurement distance to isocenter, target shape, target location, target size) are not reproduced. The estimated dose to the fetus with the Mevion single gantry PBS system is less than the reported dose for the Mevion double scattering system.^18^

## AUTHOR CONTRIBUTIONS

Karolyn M. Hopfensperger and Sara St. James led the study design and collection of proton data. Karolyn M. Hopfensperger and Xing Li led the collection of photon data. All authors participated in the analysis and interpretation of data. All authors helped write and edit the manuscript, and all authors approved of the final work.

## CONFLICT OF INTEREST

The authors report no Conflicts of Interest.

## References

[1] National Association of Proton Therapy . Accessed 07/21/2022, 2022. https://www.proton‐therapy.org

[2] Shrestha S , Newhauser WD , Donahue WP , Perez‐Andujar AA . Stray neutron radiation exposures from proton therapy: physics‐based analytical models of neutron spectral fluence, kerma and absorbed dose. Phys Med Biol. 2022;67(12):125019. 10.1088/1361-6560/ac7377 35613603

[3] Schneider U , Hälg R . The impact of neutrons in clinical proton therapy. Perspective. Front Oncol. 2015;5:235. doi:10.3389/fonc.2015.00235 26557501 PMC4617104

[4] Stovall M , Blackwell CR , Cundiff J , et al. Fetal dose from radiotherapy with photon beams: report of AAPM Radiation Therapy Committee Task Group No. 36. Med Phys. 1995;22(1):63‐82. 7715571 10.1118/1.597525

[5] Schippers JM , Lomax AJ . Emerging technologies in proton therapy. Acta Oncologica. 2011;50(6):838‐850. doi:10.3109/0284186X.2011.582513 21767183

[6] Dowdell SJ , Clasie B , Depauw N , et al. Monte Carlo study of the potential reduction in out‐of‐field dose using a patient‐specific aperture in pencil beam scanning proton therapy. Phys Med Biol. 2012;57(10):2829‐2842. doi:10.1088/0031-9155/57/10/2829 22513726 PMC3373272

[7] Hyer DE , Bennett LC , Geoghegan TJ , Bues M , Smith BR . Innovations and the use of collimators in the delivery of pencil beam scanning proton therapy. Int J Part Ther. 2021;8(1):73‐83. doi:10.14338/ijpt-20-00039.1 PMC8270095 34285937

[8] Chiang B‐H , Bunker A , Jin H , Ahmad S , Chen Y . Developing a Monte Carlo model for MEVION S250i with HYPERSCAN and Adaptive Aperture™ pencil beam scanning proton therapy system. J Radiother Pract. 2021;20(3):279‐286. doi:10.1017/S1460396920000266

[9] Olsher RH , Hsu HH , Beverding A , et al. WENDI: an improved neutron rem meter. Health Phys. 2000;79(2):170‐81. doi:10.1097/00004032-200008000-00010 10910387

[10] Zorloni G , Bosmans G , Brall T , et al. Joint EURADOS WG9‐WG11 rem‐counter intercomparison in a Mevion S250i proton therapy facility with Hyperscan pulsed synchrocyclotron. Phys Med Biol. 2022;67(7):075005. doi:10.1088/1361-6560/ac5b9c 35259730

[11] Zorloni G , Bosmans G , Brall T , et al. Eurados rem‐counter intercomparison at Maastro Proton Therapy Centre: comparison with literature data. Radiat Prot Dosimetry. 2022;198(19):1471‐1475. doi:10.1093/rpd/ncac189 36138419

[12] Kry SF , Bednarz B , Howell RM , et al. AAPM TG 158: measurement and calculation of doses outside the treated volume from external‐beam radiation therapy. Med Phys. 2017;44(10):e391‐e429. doi:10.1002/mp.12462 28688159

[13] Heimovaara JH , Blommaert J , Free J , et al. Proton therapy of a pregnant patient with nasopharyngeal carcinoma. Clin Transl Radiat Oncol. 2022;35:33‐36. doi:10.1016/j.ctro.2022.04.014 35601798 PMC9114153

[14] Knežević Ž , Stolarczyk L , Ambrožová I , et al. Out‐of‐field doses produced by a proton scanning beam inside pediatric anthropomorphic phantoms and their comparison with different photon modalities. Front Oncol. 2022;12:3386. 10.3389/fonc.2022.904563 PMC9361051 35957900

[15] Mesoloras G , Sandison GA , Stewart RD , Farr JB , Hsi WC . Neutron scattered dose equivalent to a fetus from proton radiotherapy of the mother. Med Phys. 2006;33(7):2479‐2490. doi:10.1118/1.2207147 16898451

[16] Chung CS , Yock TI , Nelson K , Xu Y , Keating NL , Tarbell NJ . Incidence of second malignancies among patients treated with proton versus photon radiation. Int J Radiat Oncol Biol Phys. 2013;87(1):46‐52. doi:10.1016/j.ijrobp.2013.04.030 23778197

[17] Hälg RA , Schneider U . Neutron dose and its measurement in proton therapy‐current state of knowledge. Br J Radiol. 2020;93(1107):20190412. doi:10.1259/bjr.20190412 31868525 PMC7066952

[18] Howell RM , Burgett EA , Isaacs D , et al. Measured neutron spectra and dose equivalents from a mevion single‐room, passively scattered proton system used for craniospinal irradiation. Int J Radiat Oncol Biol Phys. 2016;95(1):249‐257. 27084645 10.1016/j.ijrobp.2015.12.356

[19] Wang X , Poenisch F , Sahoo N , et al. Spot scanning proton therapy minimizes neutron dose in the setting of radiation therapy administered during pregnancy. J Appl Clin Med Phys. 2016;17(5):366‐376. 10.1120/jacmp.v17i5.6327 PMC5874122 27685136

